# A Pilot Study of Preparedness of Dentists in the United Arab Emirates to Deal with Medical Emergencies

**DOI:** 10.1055/s-0042-1755628

**Published:** 2023-04-14

**Authors:** Helmi Shaath, Basheer Salman, Dalia Daghistani, Rayan Koutaich, Alya Alhammadi, Nermeen Yakoub, Manal A. Awad

**Affiliations:** 1 College of Dental Medicine, Department of Preventive and Restorative Dentistry, University of Sharjah, Sharjah, United Arab Emirates; 2 Department of Oral and Craniofacial Health Sciences, College of Dental Medicine, University of Sharjah, Sharjah, United Arab Emirates

**Keywords:** dentists, medical emergencies, preparedness

## Abstract

**Objectives** The purpose of this pilot study is to assess the United Arab Emirates dentists' preparedness to manage medical emergencies.

**Materials and Methods** Ninety-seven licensed dentists participated in this study. Dentists responded to self-administrated questionnaires that contained 23 questions divided into five parts. The first part collected data on participants' sex, years of experience, and whether they are general dental practitioner (GDP) or specialists. The second part included seven questions that asked participants to indicate if they took medical history, obtained vital signs, and attended basic life support courses. The third part included six multiple choice questions regarding the availability of emergency drugs in the dental clinic. The fourth part consisted of three multiple-choice questions that assessed the dentists' immediate response to a medical emergency. Finally, the fifth part comprised four questions to evaluate the dentists' knowledge of proper treatment of special emergency cases they may encounter in the dental offices.

**Results** Out of the 97 participants, only 51% ( *N* = 49) indicated that they can handle emergencies such as anaphylactic shock and syncope in the dental office. The majority of the dentists (80%) indicated that they have emergency kits. Only 46% of the specialists and 42% of the GDPs were able to correctly plan extractions in a patient with a prosthetic heart valve. Less than half of the participants ( *N* = 35, 36%) were able to correctly answer the question regarding management of a foreign-body aspiration by attempting Heimlich/Triple maneuver.

**Conclusions** Within the limitations of this study, dentists need further hands-on training to improve their skills and knowledge about medical emergencies that could occur in the dental settings. Furthermore, we recommend that guidelines should be available in the clinic to strengthen the dentists' ability to deal with medical emergencies.

## Introduction

It is inevitable that dentists may face medical emergencies in the dental clinics. 1 Anxiety associated with dental/surgical procedures including the administration of local anesthesia can induce medically emergent situation, such as syncope, hyperventilation, airway obstruction, anaphylaxis, and possible cardiac arrest. 2 3 For well-trained dentists in diagnosing conditions and treating patients in emergencies, the availability of essential emergency drugs and equipment can reduce the risks of detrimental outcomes associated with medical emergencies. 2

Medical emergencies in dental offices are regarded as challenging worldwide, owing to the concerns about emergency preparedness, 4 5 6 7 practical skills, 6 7 as well as availability of emergency life-saving equipment and drugs. 3 8 9 10 For example, in Saudi Arabia, Jaber et al 11 reported that only 29.6% of the surveyed dentists had the essential drugs to deal with medical emergencies, while Al-Sebaei et al 12 showed that only 38.6% of the surveyed dentists had oral glucose in their clinics. Moreover, reports from Brazil 4 , Poland, 3 and Kuwait 13 point out that dentists lack competencies needed to handle medical emergencies. International guidelines call for dentists to participate in regular annual practical training in recognition and management of medical emergencies. 14 Detailed protocols are available in some countries to help prepare dentists for such situations. 15 16 For example, the American Dental Association (ADA) “Medical Emergency Manual” includes specific instructions on management of emergencies that may occur in the dental offices, such as syncope, cardiac arrest, and obstructed airway. Extensive details are also provided on procedures that should be followed in these situations.

The United Arab Emirates (UAE) is an Arabian Gulf state that consists of seven Emirates (Abu Dhabi, Dubai, Sharjah, Ajman, Ras Al Khaimah, Umm Al Quwain, and Al Fujairah) with a total population of 9.99 million ( https://www.globalmediainsight.com/blog/uae-population-statistics/ ). The UAE has four separate health authorities, the Ministry of Health and Prevention, the Health Authority-Abu Dhabi, the Dubai Health Authority, and the recently formed Emirates Health Authority. Available guidelines stipulate that each licensed dentist must have a current certificate in Basic Life Support (BLS). 17 The guidelines also include instructions that operators providing dental services shall develop policies and procedures that clearly outline the management of life-threatening emergencies and care, including cardiopulmonary, anaphylactic emergencies, and other unanticipated complications.

Although several studies have been conducted worldwide to assess the dentists' ability to deal with emergencies, 3 4 5 6 7 18 19 20 21 22 23 24 25 26 data concerning the medical emergencies in the dental offices in the United Arab Emirates are scarce.

Therefore, the aim of this pilot study is to assess the knowledge and training of dentists and the availability of medical emergency drugs in the dental offices in the UAE.

## Materials and Methods

This pilot cross-sectional study was performed between September and December 2019. The study included a convenient sample of 100 general and specialist dentists who were invited to participate in the study. The inclusion criteria stipulated that all participants must have a valid license to practice in the UAE and were at that time working in private clinics. Ethical approval was obtained from the Research Ethics Committee at the University of Sharjah (REC-18120619S-RS). All study participants read the information sheets and signed the consent forms. Participants were informed that they have the right to withdraw from the study.

The self-administered 23-item questionnaire used in this study is based on a previously used questionnaire 7 that is divided into five parts. The first part collected data on participants' gender, years of experience, and whether they are general dental practitioner (GDP) or specialists. The second part included seven questions that asked participants to indicate if they took medical history, obtained vital signs, attended BLS courses as well as their clinical ability to deal with different emergency scenarios that may occur in a dental clinic. These included their ability to give intramuscular (IM) injections and position the patient correctly in certain emergencies. For these items, responses were dichotomous (yes/no). The third part included six multiple-choice questions regarding the availability of emergency drugs in the dental clinic. The fourth part included three multiple-choice questions, which assessed the dentist's immediate response to a medical emergency. Finally, the fifth part comprised four questions to evaluate the dentist's knowledge of proper treatment of special emergency cases that could be encountered in the dental office. Based on expert opinion of one of the co-authors (B.S.) who has extensive clinical experience in the UAE, the fifth part of the survey was modified from the original questionnaire.

Two authors (H.S. and D.D.) explained the study to the participants. Each dentist was asked to complete the questionnaire using a tablet that was handed to him or her. The two authors were available to clarify any issue raised by the participants.

## Statistical Analysis

The data were entered into SPSS statistical program version 26 (IBM, Armonk, New York, NY, USA). Descriptive statistics were used to report the participants' responses; categorical variables were reported as percentages. Data from the questionnaire were organized into frequency tables. Chi-square tests were utilized to compare responses according to the dentists' status (GDP or specialist). Alpha level 0.05 (two tailed) were used for significance testing.

## Results

### Demographics of the Participants

Out of the 100 distributed questionnaires, 97 were fully completed, yielding a response rate of (97.0%), with the majority of participants being GDPs (66%). Most of the dentists encountered were males (54.3%) and more than half the participants (65%) had 6 years or more clinical experience ( Table 1 ).

**Table 1:** Characteristics of participants

Items	*N* (%)
Gender	
Female	47 (46)
Male	50 (54)
Qualification	
GP	68 (66)
MDS	29 (34)
Years of experience	
≤5 y	34 (35)
6–10 y	49 (50)
> 10 y	14 (15)

Abbreviations: GP, general practitioner; MDS, Master of Dental Surgery.

### Medical History Collection and Ability to Administer IM/IV Injections

In this study, only 32% ( *N* = 31) obtained vital signs before starting any treatment ( Table 2 ). Out of the 97 participants, 51% ( *N* = 49) indicated that they can handle emergencies, such as anaphylactic shock and syncope.

**Table 2:** Participants' responses to questionnaire items

Item	Total	GDP, *N* (%)	Specialist, *N* (%)	*p* -Value
Part 2				
1. Do you obtain medical history including medication and allergy?				
Yes	92 (95)	60 (93)	32 (97)	0.49
No	5 (5)	4 (6)	1 (3)	
2. Do you measure the vital signs (blood pressure, pulse, respiration, and temperature) before starting any treatment?				
Yes	31 (22)	23 (36)	8 (24)	0.24
No	66 (68)	41 (64)	25 (76)	
3. Have you attended any basic life support (BLS) course within the past 2 years?				
Yes	73 (75)	48 (75)	25 (76)	0.93
No	24 (25)	16 (25)	8 (24)	
4. Do you think you can handle (syncope, anaphylactic shock, foreign body aspiration, etc.) if any occurs at your dental office?				
Yes	49 (51)	34 (53)	15 (46)	0.47
No	48 (49)	30 (47)	18 (54)	
5. Are you able to administer an intramuscular injection?				
Yes	73 (75)	48 (75)	25 (76)	0.93
No	24 (35)	16 (25)	8 (24)	
6. Are you able to administer an Intravenous injection?				
* Yes*	46 (47)	31 (48)	15 (46)	0.78
No	51 (53)	33 (52)	18 (54)	
7. Have you taken any training to give intramuscular or intravenous injections?				
Yes	51 (53)	31 (48)	15 (46)	0.02
No	46 (47)	33 (52)	18 (54)	
Part 3				
8. Do you have an available emergency kit at your dental clinic				
Yes	78 (80)	48 (75)	30 (91)	0.11
No	19 (20)	16 (25)	3 (9)	
Which of the following emergency drugs are kept in your clinic?				
*9. Adrenaline*				
Yes	66 (68)	39 (61)	27 (82)	0.04
No	31 (32)	25 (39)	6 (18)	
10. Oral glucose				
Yes	67 (69)	40 (63)	27 (82)	0.05
No	30 (31)	24 (37)	6 (18)	
11. Ammonia inhalant				
Yes	30 (31)	20 (70)	44 (69)	
No	67 (69)	10 (30)	23 (70)	0.92
*12. Hydrocortisone*				
Yes	57 (59)	33 (52)	24 (73)	0.05
No	40 (41)	31 (48)	9 (27)	
*13. Atropine*				
Yes	38 (39)	21 (33)	17 (52)	0.07
No	59 (61)	43 (67)	16 (49)	

Abbreviation: GDP, general dental practitioner.

### Emergency Drugs' Availability among Dental Clinics

The majority of the dentists (80%) indicated that they have emergency kits ( Table 2 ). Compared with specialists, a significantly lower percentage of GDPs indicated that adrenaline, oral glucose, and hydrocortisone are available in their clinics ( *p* < 0.05).

### Regarding the Immediate Response in Case of Emergency

Nearly all participants (92.0%) were aware of placing the patient in the Trendelenburg position and administering ammonia inhalant in case of syncope. Less than half of the participating dentists (37.0%) knew how to manage airway obstruction due to foreign-body aspiration by immediate Heimlich/Triple maneuver. Sixty-three percent were unaware that they should activate emergency medical services after shaking and shouting an unconscious patient ( Table 2 ).

### Comparing the Knowledge between General Practitioners and Specialists Regarding Managing Special Cases

Only 46% of the specialists and 42% of the GDPs were able to correctly plan extractions in patients with a prosthetic heart valve. Nearly similar results were obtained regarding the correct anatomical area to perform chest compression during CPR (GDP [48.4%) and specialists [42.4%]) ( Table 3 ).

**Table 3:** Participants' responses to selected cases

Item	Total	GDP, *N* (%)	Specialist, *N* (%)	*p* -Value
Part 4				
What would be the correct action if a patient suffered suddenly from syncope?				
• Continue dental procedure	3	2 (3)	1 (3)	0.87
• *Place patient in Trendelenburg position and give ammonia inhalant*	91	60 (94)	31 (94)	
• Make patient to sit in upright position	3	2 (3)	1 (3)	
• Make patient stand	0	0	0	
A foreign body has been aspirated during dental treatment and caused an airway obstruction, what would be your action?				
• *Attempt Heimlich/triple maneuver*	35 (36)	24 (38)	11 (33)	0.68
• Examine mouth	5 (5)	3 (5)	2 (6)	
• Ask patient to cough	9 (9)	7 (11)	2 (6)	
• All of the above	48 (50)	30 (46)	18 (55)	
14. If you confirm somebody is not responding to you even after shaking and shouting at him/her. What will be your immediate action?				
• Start CPR	55 (57)	41 (64)	14 (42)	0.02
• *Activate EMS*	35 (36)	18 (28)	17 (52)	
• Put the patient in recovery position	2 (2)	2 (3)	0	
• Observe	5 (5)	3 (5)	2 (6)	
Part 5				
How would you plan for extraction of a tooth in a patient with prosthetic heart valve?				
• *Advise antibiotic prophylaxis and consult the patient's physician*	41 (42)	26 (41)	15 (46)	0.57
• Ask the patient to stop blood thinners	4 (4)	1 (2)	3 (8)	
• All of the above	52 (59)	37 (58)	15 (46)	
In patients with prosthetic heart valves, which of the following procedures does not require antibiotics?				
• Dental radiographs	13 (13)	9 (14)	4 (12)	0.64
• Placement of orthodontic brackets	0	0	0	
• Placement of removable prosthesis and orthodontic appliance	6 (6)	1 (2)	5 (15)	
• *All of the above*	78 (80)	54 (84)	24 (73)	
What is the location of chest compression?				
• Left side of the chest	8 (8)	4 (6)	4 (12)	0.17
• Right side of the chest	1 (1)	1 (2)	0	
• *Mid-chest*	45 (46)	31 (48)	14 (42)	
• Xiphisternum	43 (44)	28 (44)	15 (46)	
In your opinion, what is the best method to improve the dentist's knowledge and ability to manage emergency cases in the dental settings?				
• Attend more theoretical courses	2 (2)	2 (3)	0	0.06
• Attend hands on courses and workshops	63 (67)	43 (69)	20 (63)	
• Incorporate intensive medical emergency courses for undergraduate dental students curriculum	21 (22)	15 (24)	6 (19)	
• Self-study (books, journals, Youtube, online cousrses	8 (9)	2 (3)	6 (19)	

Abbreviation: GDP, general dental practitioner.

More than half of the GDPs (69%) and the specialists (63%) indicated that the best method for dentists to improve their knowledge and manage emergency cases was through attending hands-on courses and workshops.

## Discussion

In this cross-sectional study, we found that the majority of dentists (95%) obtain medical history at the initial visit. To the contrary, fewer dentists (32%) enquire about vital signs such as blood pressure, pulse, and temperature. To avoid medical complications, The ADA 15 recommends that vital signs should be assessed at the first visit of every patient and at every visit for the medically compromised patients. The observed low percentage of dentists who assessed their patients' vital signs in this study, as well as, in other studies 12 21 suggests that in general, dentists are unaware of the significance of such practice, which should be regarded essential for prompt and efficient response to a medical emergency. 16 22 For example, assessment of vital signs can provide indications of an increase in temperature, which could be due to a viral or bacterial infection. 22 Increased pulse and respiration could be due to anxiety. Accordingly, recording vital signs before a treatment will help provide baseline measurements to help monitor changes in the patient's condition.

Medical emergencies such as vasovagal reflex and hyperventilation are usually not life-threatening. However, the finding that 24% of the study dentists did not complete a BLS course within the last 2 years does not conform to the UAE guidelines that stipulate that all health care providers should have current BLS certificates. 15 This could also indicate that some dentists regard BLS skills as rarely used, because management of cardiac arrest is unlikely to occur in the dental offices. 4 10 12 Nonetheless, cardiac arrest is the most critical threat to life, and dentists are advised to obtain BLS-related knowledge and skills at least every 2 years. 12

Despite the clear recommendations from the UAE Ministry of Health and Prevention that dentists should have emergency kits available, our findings show that 25% of the GDPs and 9% of specialists did not have emergency kits in their dental offices. These percentages are similar to those reported in Saudi Arabia 11 and Germany, 18 and much lower than those reported in India 26 and Iran. 27 However, it should be emphasized that medical emergency can occur and cause death of the patients if not handled appropriately. 13 14 For example, syncope which could occur due to phobia or hypoglycemia is not specifically considered as an “emergency,” however, if not handled appropriately, could lead to an emergency such as cerebral ischemia. 14 The most commonly available emergency drugs in the emergency kits were oral glucose and adrenaline (69 and 68%, respectively). Similar to previous reports, 12 22 the least available drugs were atropine (38%) and ammonia inhalants (31%). These findings suggest that dentists are unaware of the current recommendation by the UAE health authorities to include atropine in the emergency kit, 28 or with suggestions to use ammonia inhalants as a respiratory stimulant in dentistry. 29 30

Consistent with previous reports, 12 22 31 a relatively high percentage of the participants (53% of GDPs and 46% of specialists) indicated that they would not be able to handle emergencies in the clinic. In our study, 50% of the dentists admitted that they could not administer IM injections and 25% could not administer intravenous (IV) injections, both may be needed in case of an emergency. However, it is worth noting that administration of IV by dentists is a controversial issue. 32 Some recommended that dentists should be able to administer drugs intravenously. 33 34 While others advocate against the use of IV route in an emergency, because dentists are not critical care providers. Moreover, grasping this technique is difficult, since most dental schools do not train their graduates on this procedure. 32 Nonetheless, the ability to administer IV drugs may be inevitable in case of a cardiac arrest.

Most of our participants (91.0%) knew how to deal with a patient who has syncope; this is not surprising, since syncope is the most prevalent emergency that occurs in the dental offices. 1 19 21 To the contrary, fewer dentists were able to manage correctly foreign-body aspiration by attempting Heimlich/Triple maneuver. Although it is a rare occurrence in the dental setting, knowledge about management of such a life-threatening medical emergency should not be disregarded. 35

Despite the commonality of antibiotic prophylaxis use in dental practices, the understanding of recommended guidelines for its use in some patient groups is deficient, 36 37 as knowledge related to conditions where prophylaxis is indicated varied widely amongst participating dentists. 37 Our findings show that only 42% of the dentists chose to recommend prophylactic antibiotics in the case of extraction of a tooth in a medically compromised patient. The current guidelines from the health authorities in the UAE support this practice. 38 Accordingly, there is a necessity to reinforce the current guidelines.

Attending hands-on courses and workshops was the most popular selected method for gaining skills and knowledge regarding medical emergencies. Accordingly, the health authorities could consider this preferred method as a means to contribute to the delivery of safer dental services in the UAE. Moreover, in light of the current COVID-19 (coronavirus disease 2019) pandemic, planning such courses should include being prepared to respond to a cardiac arrest in a patient with COVID-19 or suspected to have COVID-19. 39

This pilot study provides important preliminary data on emergency management skills of the dentists in the UAE. However, our study has certain methodologic limitations. This research was designed as a pilot study, allowing for a relatively small sample size. Better representation from all the emirates should be considered in future studies. The questionnaire used in this study is based on a previous study among dentists in a hospital facility, not in the private practice settings. It should also be noted that many dentists who practice in the UAE graduated from different universities worldwide, in which teaching management of medical emergencies may vary by institute. Although this could affect the results of this study, the majority of the surveyed dentists (65%) have been working in the UAE for more than 6 years, ample time to be familiar with the UAE guidelines regarding management of medical emergencies. Nevertheless, we recommend the development of a national protocol to regulate the management of medical emergencies in dental practices, particularly the non-hospital-based private dental practices, as well as to ensure the availability of recommended emergency drugs.

## Conclusion

Our findings highlight the relevance and importance of dentists' preparedness to handle medical emergencies. Our results show that dentists need additional training on handling such emergencies. We also found a deficiency in the availability of drugs and emergency equipment among the surveyed dentists. For effective management of medical emergencies in the dental clinics in the UAE, regulations for emergency preparedness must me reinforced by the health care authorities.
